# Cryoablation for fibroadenoma with liquid nitrogen based system: A retrospective analysis of prospectively collected data

**DOI:** 10.1371/journal.pone.0340969

**Published:** 2026-01-23

**Authors:** Teodóra Filipov, Brigitta Teutsch, Dorina Vass, Boglárka Budinszki, Péter Hegyi, Attila Doros, Gábor Forrai, Pál Ákos Deák

**Affiliations:** 1 Premier Med Institute for Health, Education, and Research, Budapest, Hungary; 2 Department of Interventional Radiology, Faculty of Medicine, Semmelweis University, Budapest, Hungary; 3 Centre for Translational Medicine, Semmelweis University, Budapest, Hungary; 4 Medical Imaging Centre, Faculty of Medicine, Semmelweis University, Budapest, Hungary; 5 Institute for Translational Medicine, Medical School, University of Pécs, Pécs, Hungary; 6 Institute of Pancreatic Diseases, Semmelweis University, Budapest, Hungary; Chongqing Medical University, CHINA

## Abstract

**Background:**

Fibroadenoma is the most common benign breast lesion found on core needle biopsies. Surgical excision is the standard of care for these lesions. This retrospective study aims to evaluate the safety and efficacy of liquid nitrogen-based cryoablation in treating multiple fibroadenomas, including large lesions.

**Methods:**

A liquid nitrogen-based cryoablation system was used to treat histologically confirmed benign fibroadenomas under ultrasound guidance at Premier Med Healthcare, Training, and Research Institute between 2017 and 2022. The number and times of freeze-thaw-freeze treatment cycles and the number of cryoprobe relocations were determined according to the location and size of the fibroadenomas. Sequential cryoprobe relocation was performed in case of large or multiple fibroadenomas treated in one session. Patients underwent ultrasound examination follow-up visits for up to 12 months post-cryoablation. Data were analyzed descriptively. Changes in lesion size were evaluated using the Wilcoxon signed-rank test. A p-value < 0.05 was considered statistically significant.

**Results:**

78 women with a mean age of 34.2 ± 9.8 were included. The number of lesions per patient ranged from 1 to 4, with 60% having one lesion, 25% two, 13% three, and 3% four. Lesions were evenly distributed between the left (48.4%) and right (51.6%) breasts, with the upper outer quadrant being the most common location (28%). Lesion size, diameter of the largest dimension, ranged from 7 to 80 mm (mean 25 ± 10.9). The mean procedure time was 13 ± 10.4 minutes with 1−5 relocations per cryoprobe. In 76% of cases, a single freeze-thaw-freeze cycle was sufficient. Multiple cryoprobe relocations were used for larger or multiple lesions to ensure full coverage. The median volume reduction was 80.6% (IQR: 56.6–92.6) at 6 ± 1.5 months and 92.9% (IQR: 73.6−100) at 12 ± 1.5 months. The reduction observed at 12 months (mean follow-up of 16.3 ± 10 months) was statistically significant (p < 0.0001). One minor adverse event occurred (1/123 = 0.81% [95%CI: 0.02%−4.45%]) that resolved with conservative treatment.

**Conclusion:**

Cryoablation with a liquid nitrogen-based system proved safe and effective, with 92.9% volume reduction at one year post-cryoablation for fibroadenoma. Sequential cryoprobe relocations preserve safety and efficacy, allowing the flexibility necessary for complete ablation of large or multifocal lesions. With the inclusion of a large patient cohort, our study further reinforces the clinical value of cryoablation and brings the technique one step closer to integration into routine practice.

## Introduction

According to the Community Research and Development Information Service, an estimated 1.3 million breast biopsies occur annually in Europe [1]. The majority of them reveal benign breast disease. With an incidence of 25%, fibroadenoma (FA) is the most common benign breast lesion generally found before the age of 30 in females in the general population [2].

Fibroadenomas (FAs), though benign, can still cause psychological distress, with patients often worrying about misdiagnosis, potential malignancy, or discomfort when touching the lump [3]. Standard of care options for symptomatic benign tumors, including fibroadenomas, include Surgical excision (i.e., lumpectomy, excisional biopsy, or vacuum-assisted excision (VAE)) or observation. However, minimally invasive techniques, such as thermal ablation, now provide additional alternatives for FA management [4–7].

The only thermal ablative technique to use cooling elements (such as liquid nitrogen) is cryoablation (CA), which is approved by the United States Food and Drug Administration [7] and the European conformité européenne (CE) for the treatment of FA [8]. CA offers several advantages, including (1) excellent visualisation of the ice ball under ultrasound (US) as compared to heat-based ablation, permitting precise procedural control and skin protection; (2) minimal to no scarring, as compared to surgery; (3) May be performed under local anaesthesia; (4) patient can resume her everyday life almost immediately; and (5) an ideal office-based procedure [9].

CA has been effectively utilized to treat both malignant and benign conditions. In the management of breast fibroadenomas (FAs), studies have reported that CA is a safe and effective approach, with positive outcomes observed in both short- and long-term follow-ups [10], as well as high satisfaction levels among patients and physicians [11]. The current study aimed to further support existing data on the effectiveness and safety of cryoablation and demonstrate the feasibility of cryoprobe relocation method that allows the treatment of large and/or multiple FAs in a single session through multiple treatment cycles.

## Materials and methods

### Study design and setting

This study was approved by the local ethical institutional review board, License number: 70/2024; BP-02/NEO/39703–3/2021, Professional code: 5206, Organizational unit identifier: 001076581. All procedures performed were in accordance with the ethical standards of the institutional research committee and with the 1964 Helsinki Declaration and its later amendments or comparable ethical standards. Written informed consent was obtained from all individual participants included in the study.

This is a retrospective single-centre observational study with the primary objective of demonstrating the efficacy of CA for FA. The study was structured and reported in accordance with the Strengthening the Reporting of Observational Studies in Epidemiology (STROBE) Statement guidelines (S1 Table in S1 File) to ensure clear and comprehensive reporting. A secondary aim is to demonstrate that CA is safe for treating FA in general and when using the cryoprobe relocation technique.

Procedures and data acquisition were performed at Premier Med Healthcare, Training and Research Institute, Budapest, Hungary, a private institute treating FAs with liquid nitrogen-based CA. All CA procedures performed at the centre between 2017–2023 were consecutively included, provided the FA had been confirmed by core needle biopsy. Data were accessed for research purposes through June 1^st^, 2024. The authors had access to information that could identify individual participants during and after data collection.

### US-guided cryoablation procedure

**Fig 1:**
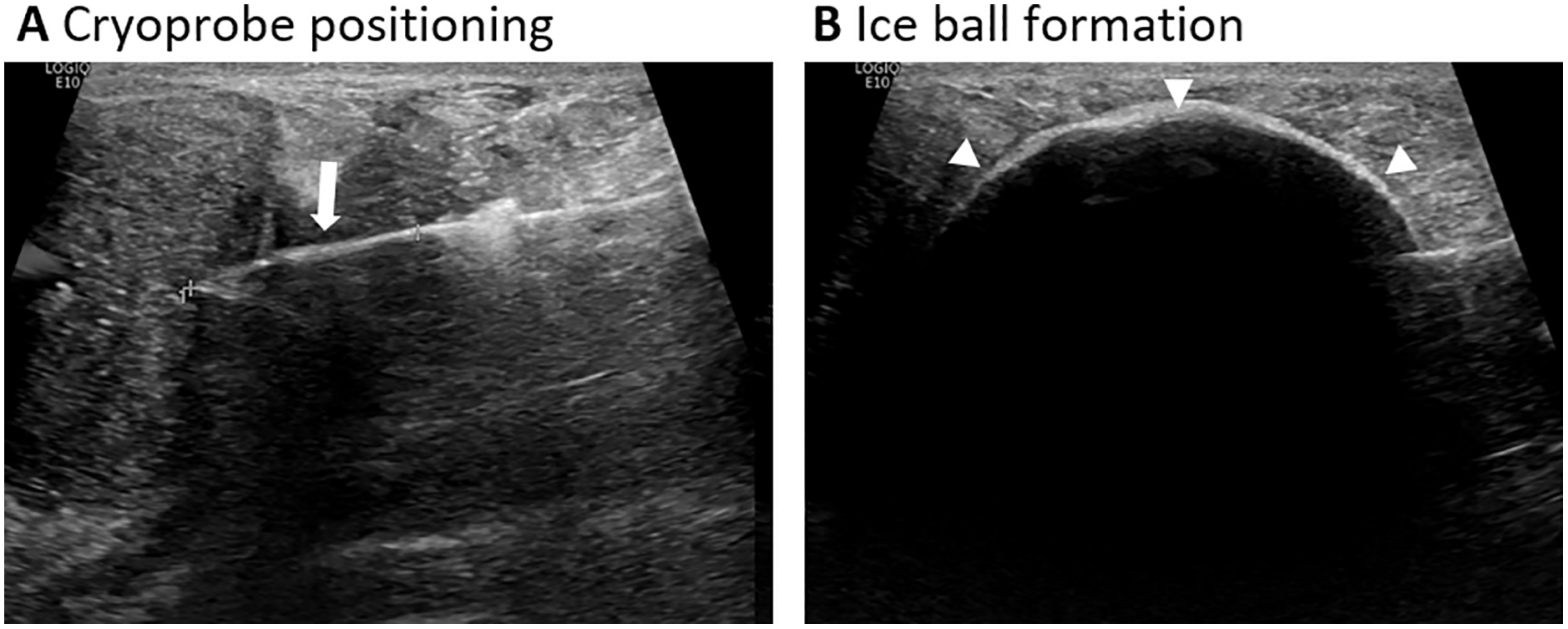
Procedure real-time images. US-guided cryoprobe (white arrow) centring within the FA **(a)** and subsequent ice ball (arrowhead) formation **(b)**.

All patients were treated with CA due to their preference and were found suitable for the procedure; they approached Premier Med Healthcare, Training, and Research Institute because they declined surgical excision, the standard of care offered to FA patients in Hungary [12]. CA procedures were performed under US guidance (Fig 1) using the ProSense^TM^ liquid nitrogen-based CA system and cryoprobes (IceCure-Medical LTD., Israel). Cryoprobe (FAP7800000 2.4 mm, 13G, 134 mm, Elliptic ice ball shape); FAP7100000 3.4 mm, 10G, 127 mm, Spheric (ice ball shape); or FAP7200000 3.4 mm, 10G, 140 mm, Elliptic ice ball shape)) and cycles duration were selected by the physician based on the size, number, accessibility, and anatomical location of the FA while referring to the manufacturer’s User Manual [13].

The 2.4 mm (13G) probe was useful for smaller fibroadenomas or when a narrower diameter reduced tissue trauma at insertion. The 3.4 mm (10G) probes generated larger ice-balls, making them suitable for medium to larger lesions. Differences in probe length (127 mm vs. 134–140 mm) allowed the physician to reach lesions at varying depths while maintaining accurate placement of the cooling zone at the lesion center. In addition, the choice between spherical and elliptical ice-ball shapes provided flexibility to match lesion geometry, helping ensure that the ice-ball conformed to the fibroadenoma’s dimensions for complete coverage. Freeze-thaw-freeze cycle duration was then tailored to achieve full lesion coverage, monitored in real time under ultrasound.

When the ice ball was not adequately engulfing the lesion (e.g., in cases of large FAs), the cryoprobe was repositioned sequentially after each freeze-thaw-freeze cycle to achieve full engulfment.

All patients received local anaesthesia (1% lidocaine, 10 ml) before the intervention. When the FA was too close (1 cm) to the skin, saline (10–50 ml) hydro dissection was used to prevent frostbite. None of the patients underwent excision after CA.

### Outcome measures

FA dimensions pre- and post-CA were determined under US imaging, and the volume was calculated as [$\frac{\pi }{\text{6}}\times length\times width\times diameter\;$(cm^3^)]. CA freeze-thaw-freeze cycle time and complications (during and up to 3 days post-CA) were recorded. Follow-up visits were planned at 1, 3, 6, and 12 months.

### Statistics

For the statistical analysis, two analysis sets were determined:

*Safety Analysis Set (SAS)*- includes all patients who underwent CA procedure (adverse events during and up to 3 hours following the procedure); *Analysis Set (AS)*- consists of all patients who underwent CA procedure without major protocol deviations and had at least one follow-up during the 6 months (±1.5 months) or the 12 months (±1.5 months) post-CA. Incomplete treatment, missing follow-up, or follow-up time shorter than 1.5 months were considered major deviations.

Continuous variables were presented with mean, standard deviation (SD), median and range. Categorical variables were presented as counts and percentages. The percentage of procedure related adverse events was presented with 95% confidence intervals (95%CI).

For each lesion and at each follow-up visit, the precentage of reduction in lesion size was calculated relative to baseline. The significance of the percent reduction at 6- and 12-months follow-up was tested with Wilcoxon signed-rank test.

Subgroup analyses were carried out for probe-type, using the Kruskal-Wallis test and for age and BMI, using the Spearman correlation. A p-value<0.05 was considered statistically significant for all test performed.

Statistical analyses and data management were performed using SAS 9.4 software (SAS Institute Inc., Cary, NC, USA).

## Results

### Patient and FA characteristics and procedure

**Fig 2:**
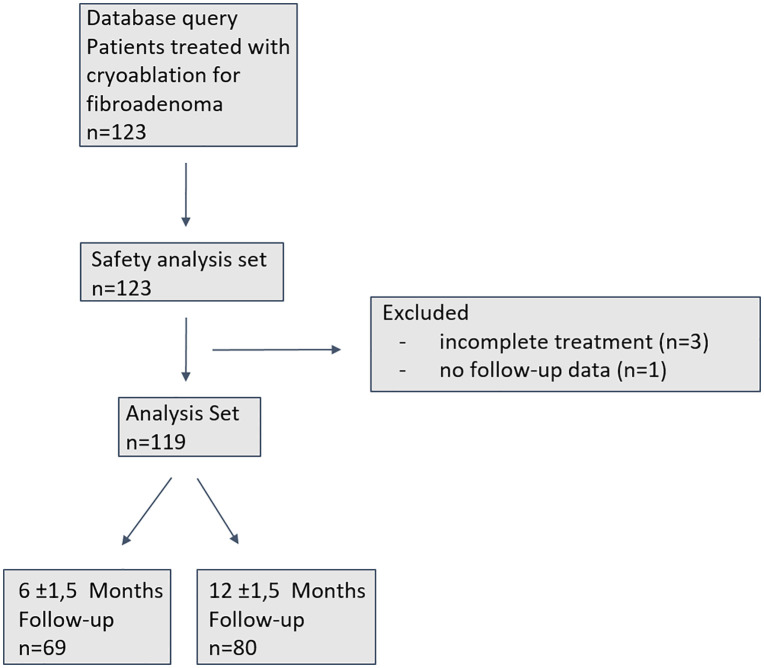
Patient’s flow *(n* number of fibroadenomas).

In total, 78 patients with 123 FAs underwent CA procedures. Cases with detected lump post-treatment were followed and monitored regularly. None of those lesions grew; therefore, none of the patients underwent excision after CA. The patient’s flow is depicted in Fig 2.

Patients’ mean and median age were 34.2 (SD ± 9.8) and 31.5 (IQR 26–34) years, respectively, ranging from 17 to 66, with the majority (62%) 35 years old or younger. The mean Body Mass Index (BMI) was 22.3 ± 4.5, with most women (81.7%) having a BMI < 25 (Table 1).

**Table 1:** Patient characteristics.

Parameter		SAS	AS
**Age** (years)	N	78	76
	Mean ± SD	34.2 ± 9.8	34.3 ± 9.9
	Min, Max	17, 66	17, 66
	Median [IQR]	31.5 [26, 43]	32.5 [26, 43]
**Weight** (kg)	N	60	58
	Mean ± SD	61.8 ± 10.7	61.7 ± 10.8
	Min, Max	42, 100	42, 100
	Median [IQR]	61 [54, 66]	61 [54, 66]
**BMI**	N	60	58
	Mean ± SD	22.3 ± 4.5	22.3 ± 4.5
	Min, Max	16, 37.8	16, 37.8
	Median [IQR]	21.9 [19.1, 23.9]	21.8 [19.1, 23.9]
**BMI < 25**	N	49	47
	%	81.7	81.0

*SAS* Safety Analysis Set; *AS* Analysis Set; *N* number of patients with available data; *SD* standard deviation; *Min* minimum; *Max* maximum; *IQR* interquartile range; *kg* kilogram; *BMI* body mass index.

The number of FAs per patient ranged from 1 to 4. The majority, 85%, had 1 or 2 FAs, with 60% having only 1 and 25% having 2 FAs (Table 2).

**Table 2:** Number of FAs per patient.

Number of FAs	Number of Patients (%)	Total Number of FAs
1	47 (60)	47
2	19 (25)	38
3	10 (13)	30
4	2 (3)	8
**Total**	**78**	**123**

*FA* fibroadenoma.

**Fig 3:**
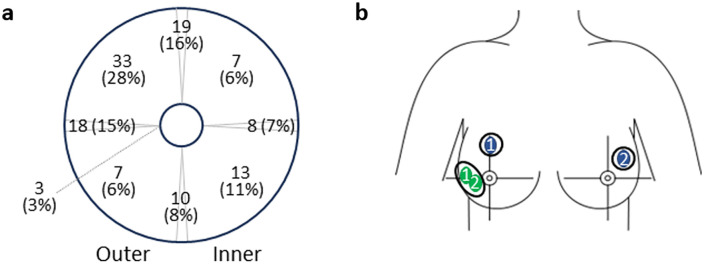
Fibroadenoma (FA) localisation. Distribution of the different FAs by side (outer/ inner) and quadrant location **(a)**. Two examples (depicted on the same fig), one of a patient with 2 FAs in close proximity in the left breast (green circles marked 1 and 2) and one of a 48-year-old patient with 2 FAs, one in the left breast and one in the right breast (blue circles marked 1 and 2) **(b)**.

FAs were equally distributed between the left and the right breast, while the upper outer quadrant was the most common location (28%) (Fig 3a).

FAs’ size (as determined by the diameter of the largest dimension) and volume varied significantly: the diameter ranged from 7 mm to 80 mm (mean 25.0 ± 10.8), and the volume ranged from 0.1 to 62.8 cm^3^ (mean 5.7 cm^3^ ± 7.9 cm^3^).

In most procedures (90.2%), a 3.4 mm (10G) cryoprobe was used, as recommended by the manufacturer (for more details see Methos section), with a mean procedure time, freeze-thaw-freeze cycle, of 13.0 ± 10.4 minutes (Table 3).

**Table 3:** FA characteristics at baseline and procedure features.

Parameter			SAS N = 122	AS N = 119
**FA**				
**FA size**	**Width**	Mean ± SD	25 ± 10.9	24.8 ± 10.7
	(mm)	Min, Max	7, 80	7, 80
		Median [IQR]	25 [16, 32]	25 [16, 31]
	**Height**	Mean ± SD	19.7 ± 8.6	19.6 ± 8.6
	(mm)	Min, Max	6, 50	6, 50
		Median [IQR]	19.5 [13, 25]	19.0 [13, 25]
	**Depth**	Mean ± SD	14.2 ± 7.4	13.8 ± 6.5
	(mm)	Min, Max	3, 54	3, 38
		Median [IQR]	13 [9, 18]	13 [9, 18]
	**Volume**	Mean ± SD	5.7 ± 8.0	5.4 ± 7.5
	(cm^3^)	Min, Max	0.1, 62.8	0.1, 62.8
		Median [IQR]	3.5 [0.9, 7.4]	3.3 [0.9, 7.2]
**FA location**		Left breast	59 (48.4%)	58 (48.7%)
		Right breast	63 (51.6%)	61 (51.3%)
**Procedure**				
**Cryoprobe diameter**		2.4 (13G)	12 (9.8%)	12 (10.1%)
(mm)		3.4 (10G)	110 (90.2%)	107 (89.9%)
**Probe length**		127 (10G)	70 (57.4%)	68 (57.1%)
(mm)		134 (13G)	12 (9.8%)	12 (10.1%)
		140 (10G)	40 (32.8%)	36 (32.8%)
**Cryoablation time**		N	116^1^	112^1^
(minute)		Mean ± SD	13.0 ± 10.4	12.8 ± 9.9
		Min, Max	3.3, 56	3.3, 56
		Median [IQR]	9 [6, 15]	9 [6, 15]
**Follow up time**		N	123	119
(months)		Mean ± SD	16.3 ± 10.0	16.2 ± 10.0
		Min, Max	1.1, 42.2	1.1, 42.2
		Median [IQR]	13.0 [11.7, 23.3]	12.9 [11.7, 23.3]

^1^For 5 subjects, the cryoablation procedure was done on two FAs during the same procedure. Therefore, the procedure time for these cases was only considered once.

*SAS* Safety Analysis Set; *AS* Analysis Set; *N* number of patients; *SD* standard deviation; *IQR* interquartile range; *Min* minimum; *Max* maximum; *kg* kilogram; *cm* centimeter.

### Number of freeze-thaw-freeze cycles per procedure

In most cases (76%, 91/119), one freeze-thaw-freeze cycle was used per FA. The same methodology was used when the number of lesions was > 1, but the lesions were close to each other (≥5 mm apart). In the rest of the cases, one or more relocations were performed by (partly or entirely) removing and repositioning the cryoprobe for additional freeze-thaw-freeze cycles. Relocations were applied when 1) there were two or more FAs distant from each other, 2) the FA was large, and 3) the FA location did not allow treatment in one cycle. For example, in two FAs > 43mm cases in the largest dimension (one 48x20x27 mm and the other 80x50x30 mm), 3 and 4 cycles were required for complete ablation.

An example of US imaging findings for a patient with two FAs in the right breast, 44 mm and 42 mm in their largest dimension (combined largest diameter of 64 mm), is displayed in S1 Fig in S1 File. Since the FAs were in close proximity (Fig 3b (green circles)), they were treated together. Three cycles, 8-8-8, 8-6-6, and 4-4-4 minutes, with three relocations, were performed with no complications reported. Volume reduction at 6 months (S1b Fig in S1 File) and 12 months (S1c Fig in S1 File) were 85%/92% and 93%/ 94%, respectively, with no complications reported.

For another patient, a 48-year-old woman with 2 FAs, one in the right breast (38 mm in its largest dimension) (Fig 3b, blue circles) and S2a–S2c Fig in S1 File) and one in the left breast (13 mm in its largest dimension) (Fig 3b, blue circles) and Fig S2d–S2f in S1 File), two sequential CA cycles were assigned (1 cycle/ FA/ breast). The total procedure time was 30 minutes, and no complications were reported. Lesion volume was reduced at 6 months (S2b and S2e Fig in S1 File) and continued to decrease at 12 months (S2c and S2f Fig in S1 File) to 96% and 57%, respectively.

### Efficacy- Volume reduction

**Fig 4:**
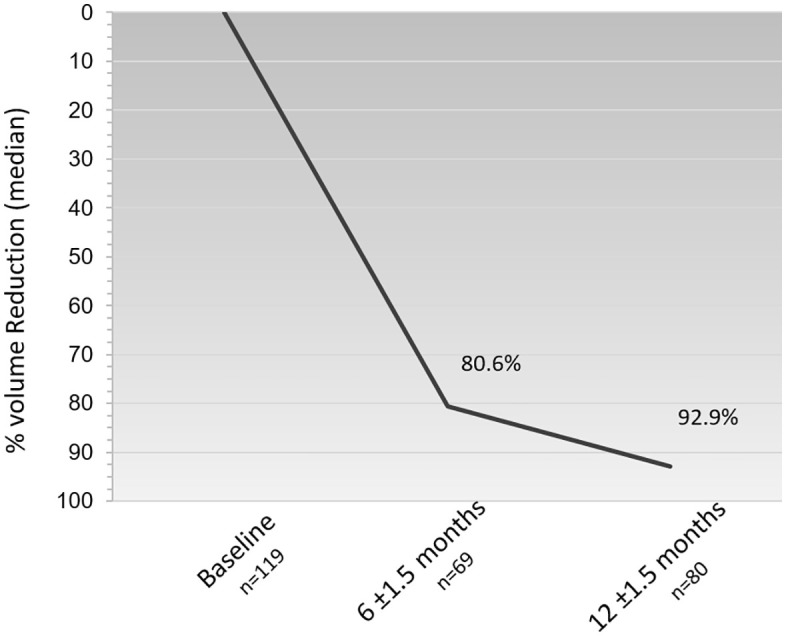
Median percent FA volume reduction.The median percentage volume reduction of FA at 6 ± 1.5 and 12 ± 1.5 months was calculated for all FAs that had data at either time point (see Methods for details).

At 12 months follow-up (mean of 16.3 ± 10 months), a significant median reduction of 92.9% (IQR: 73.6–100, p-value < 0.0001) in FA volume was observed for patients whose information was available. The decrease was gradual, with a sharp reduction in volume at the 6-month follow-up (median 80.6% [IQR: 56.6–92.6]) and a more moderate reduction up to the 1-year follow-up (Fig 4). Analysis was performed in all cases, with any initial volume/FA size, and included FAs with the largest dimension of 80 mm and volume up to 62.8 cm^3^.

### Efficacy- Volume reduction – subgroups analyses

Subgroup analyses were performed to evaluate whether patient, lesion, or procedural characteristics influenced the percentage of fibroadenoma volume reduction at 12 months. The variables tested included age, BMI, lesions (>4 cm), and cryoprobe type.

None of these factors demonstrated a statistically significant association with treatment response, suggesting that cryoablation efficacy was consistent across different patient characteristics, lesion sizes, and cryoprobe type.

Spearman’s correlation coefficients for age and BMI were non-significant (p-value = 0.69 and p-value = 0.46, respectively). According to the Kruskal–Wallis test, there was no statistically significant difference in 12-month volume reduction among the three cryoprobe types used in the study (p-value = 0.6831).

**Fig 5:**
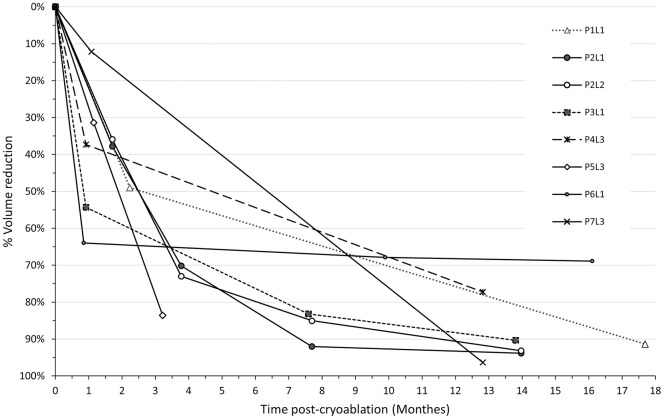
Volume reduction for lesions larger than 40 mm. The graph presents the percentage volume reduction over time following cryoablation of eight fibroadenomas (FAs) in seven patients. Each line represents an individual FA with available follow-up measurements. Volume reduction was calculated at each time point where post-procedural data were available (see Methods for more details).

Seven patients had lesions (total 8 lesions) larger than 4 cm in their largest dimension. Those patient ages ranged from 17 to 45 years with FAs lesions from 41 to 80 mm in size. Three patients had one lesion larger than 4 cm each (P1, P3, P6), one patient (P2) had two lesions >40 mm, and 3 patients (P4, P5, P7) had 2 additional lesions each, smaller than 40 mm (Table 4). As observed with smaller lesions, the volume of these large lesions decreased over time post-cryoablation, with an initial sharp reduction followed by a more gradual during extended FU (Fig 5). The mean and median reduction rates were 87 ± 1% and 91% (IQR, 82%−91%), respectively, at a mean FU of 12.9 ± 4.6 months (range, 3.2–17.7 months). Lesion 3 (L3) of patient 5 (P5) exhibited a sharp linear reduction in volume, reaching 84% at 3.2 months. However, this patient was lost to follow, and its final reduction remains unknown.

**Table 4:** Characteristics of patients with a lesion(s) larger than 40 mm.

Patient (P) number	Age (Years)	Number of lesions	Lesion (L) number and size* (mm)	Lesion Location	Number of cycles	Total Cryoablation time (min)
**P1**	26	1	L1, 45	UOQ	2	15
**P2**	17	2	L1, 42	9:00	3	56
			L2, 44	9:00	3	
**P3**	45	1	L1, 80	UOQ	3	24
**P4**	42	3	L1 22	9:00	1	51
			L2, 32	6:00	1	
			L3, 41	12:00	1	
**P5**	38	3	L1, 16	LIQ	1	36
			L2, 28	6:00	1	
			**L3, 42**	LIQ	1	
**P6**	30	1	**T1, 48**	UIQ	3	33
**P7**	34	3	T1, 21	LOQ	1	48
			T2, 21	9:00	1	
			**T3, 43**	9:00	1	

*UOQ* Upper Outer quadrant; *UIQ* Upper Inner Quadrant; *LOQ* Lower Outer Quadrant; *LIQ* Lower Inner Quadrant; *mm* millimeter; *min* minute.

* In the largest dimension.

### Safety

One adverse event was reported (1/123 = 0.81% [95%CI: 0.02%−4.45%]), a minor skin burn that resolved with conservative treatment (warming pads) with no other intervention necessary. Three incomplete treatments were recorded (3/125, 2.4%): One was due to a technical limitation (probe malfunction, that lest a 2 mm peripheral zone untreated; One due to the lesion’s proximity to the nipple, leaving a 1 mm peripheral zone untreated; and one involving a patient who had previously undergone a procedure for two FAs. At the 1 year follow-up, a second CA procedure was performed to treat residual viable (vascularised) parts of the lesions in the medial (12x23mm) and caudo-lateral (13x7 mm) regions as observed by US. Consequently, the complete treatment rate was 97.6% (122/125).

## Discussion

In this first CA for breast FA retrospective single-centre study in Hungary, the volume reduction and safety of CA for the treatment of FAs in a diverse population (n = 78) in terms of age (17–66 years old), number of FAs (1–4 FAs), and FA size 7–80 cm (volume, 0.1–62.8 cm3) were assessed.

The procedure, under local anaesthesia, was short (mean 13.0 ± 10.4 minutes per freeze-thaw-freeze cycle), and all patients were discharged on the same day and were able to resume normal activities immediately after the procedure. Although some procedures were performed with sequential (up to 5) relocations in the same session, only one (1/123 FAs, 0.82% of cases) adverse event (non-serious, minor) was reported, demonstrating a lower complication rate compared to surgical excision. For example, Fine et al [14] reported a rate of 0.93% (2/216) serious adverse events (SAE) following VAE and another study reported 6% (7/110) non-serious AE among women with FAs smaller than 3 cm, (all minor complications involving immediate post-excision hematoma) [15]. Aesthetic outcomes were well preserved with 0% unfavorable aesthetic changes. Additionaly, in the current study, the complete treatment rate (97.6%) was comparable to or higher than the complete excision rates reported after VAE (94.7% and >94%, respectively [16]).

Aesthetic outcomes were well preserved, with no cases of unfavorable aesthetic change (0%).

### Efficacy

In our study, a 92.9% volume reduction (median) was achieved at 12 months (± 1.5 months) for FAs with the largest dimension of 80 mm or smaller at baseline and 91% for FAs with the largest dimension between 40 and 80 mm. Age, BMI, and cryoprobe type did not show any correlation with the degree of fibroadenoma volume reduction, suggesting uniform treatment response across the study population.

Previous studies suggest that FAs measuring less than 20 mm responded best to CA, while larger FAs measuring more than 25 mm before CA have a greater likelihood of residual tissue at 6- and 12-month follow-ups [10]. It is postulated that since the larger FAs may have a slower rate of post-CA volume reduction, a longer follow-up was required to observe complete regression in those studies. For FA < 3 cm3 in size, Hahn et al [11] reported that the reduction rate was 73% at 6 months and 75% at 12 months, while in Golatta et al [5], lesions 0.12–5.1 cm3 in size had entirely disappeared in 93% of cases at 12 months.

The volume reduction at 6 months post-CA in the current study is higher than the range reported in the literature and in accordance with studies available in the literature for 12 months using a liquid nitrogen-based CA system. Nevertheless, comparisons across studies remain difficult due to differences in post-CA assessment (imaging vs histology), lesion size distribution, and cohort size. Other factors, such as the number of freeze cycles and relocations, ice ball size, and type of cryoprobe are additional factors that may influence the efficacy [10]. Therefore, each case was discussed in a multidisciplinary meeting, allowing individual tailoring of these parameters, which likely contributed to the high efficacy observed.

### Technical and anatomical considerations

Breast size and location of the FA within the breast tissue are critical factors that influence the technical success and safety of CA. A safe distance from the skin surface and chest wall should be maintained to prevent frostbite [17,18]. FAs closer to the nipple may be more challenging to treat technically due to concerns about damaging nipple function and nipple sensation. Our experience shows that CA can be safely utilised to treat this location. Furthermore, the tissue composition of the breast may also play a role, presenting challenges in cryoprobe positioning, particularly in denser tissue [19].

### Advantages and patient experience

Treatment for FAs may be indicated for patients with symptoms such as pain, palpability, and/or growth and include hormone therapy, surgical excision (including VAE), and US-guided CA. The American Society of Breast Surgeons Oct. 2018 Guidelines found CA of FAs to be similar in efficacy and safety to open surgical excision, approving the use of CA for FAs < 4 cm in their larger diameter [7]. CA for breast tumors (including FAs and breast cancer) is cosmetically advantageous since the treatment does not require the removal of normal surrounding breast tissue, and there is restoration of normal or near normal breasts. Similar to the current study, in recent studies of CA for BC, patients reported 100% satisfaction post-CA procedure [20–22] compared to 73.9%, 19.6% and 6.5% reporting good, intermediate, and fair satisfaction, respectively, after VAE [16]. In addition, the procedure is considerably shorter (mean of 13.0 ± 10.4 minutes per CA cycle in our study) and can be performed office-based with local anaesthesia instead of general anaesthesia [20,23]. This is especially beneficial for women with multiple FAs [5]. Finally, CA procedures also have a financial advantage over surgical excision; with an estimated 500,000 FAs surgically excised every year, the cost of surgical excision in a community hospital is approximately $18,000 versus $3,500 for cryoablation [11].

### Radiologic assessment and follow-up

The use of ultrasound (US) for post-cryoablation assessment necessitates distinguishing between radiologic endpoints (volume reduction) and pathologic endpoints (cellular viability). All treated lesions were histologically confirmed as benign FA through pre-treatment core needle biopsy, consistent with clinical guidelines [7]. Given the benign nature and predictable involution of these lesions, post-treatment excision to verify complete necrosis would be ethically and practically unwarranted. Although US-based volume measurement serves as an indirect indicator of treatment efficacy, its role as a surrogate endpoint for long-term monitoring is well established [24]. Any potential imaging bias, such as missing rare residual viable cells within the involution zone, is mitigated by the initial benign diagnosis and the proven safety of the procedure. In clinical practice, unexpected imaging findings following treatment, including regrowth or atypical morphology, would appropriately trigger re-biopsy to confirm radiologic-pathologic correlation. Accordingly, in biopsy-proven benign FAs, US-based volumetric surveillance remains a safe, practical, and widely accepted approach for long-term follow-up.

### Strengths and limitations

To the best of our knowledge, the current study includes one of the largest cohort reported from the central European area. Additionally, it demonstrated that the relocation of cryoprobes, a technique not widely described in many studies, is safe and effective for treating larger or multiple FAs. Lastly, the population was highly diverse in terms of age, number, and size of the FA, which is representative of the diversity of real-life situations. This study has some limitations, including being a single-centre study and the absence of a control group. The patient population is limited to those who preferred CA; therefore, the results may not be generalisable to all patients with FA. Additionally, because of the retrospective design of this study, some data was not captured (e.g., palpation; however, its results are inherently non-standardizable).

### Implication for practice and research

Given its low cost, high safety, and proven effectiveness, CA warrants wider adoption in clinical practice provided it is performed by experienced interventional radiological. Extensive studies comparing CA with control groups would be an option to strengthen the evidence on FA CA. Still, because of the highly dissimilar nature of interventional and surgical treatment, these types of studies might be more challenging to carry out. While CA is an accepted (US FDA-approved) method in benign cases (for core-needle biopsy confirmed FA), in Europe and Hungary, it is not yet funded by the National Health Insurance Fund of Hungary [8]. We hope that our efficacy and safety profile data will add to the body of evidence that will lead to its inclusion in insurance funds.

## Conclusion

In summary, this study provides additional evidence that CA with a liquid nitrogen system is safe and effective treatment for biopsy-proven breast fibroadenomas, with a 92.9% volume reduction at 1-year post-CA. The results suggest that the population for which cryoablation is safe and effective may be broader, including patients with large or multiple fibroadenomas. They also show that multiple sequential probe relocations do not compromise safety while maintaining effectiveness. The procedure is well tolerated, minimally invasive, and allows for rapid patient recovery, making it a practical alternative to surgical excision in the outpatient setting. By presenting real-world data from a diverse patient population, this study supports the potential for wider adoption of cryoablation in the management of benign breast lesions. The promising results of this study warrant further prospective, controlled trials to validate and expand upon the findings.

## Supporting information

S1 File Supplementary material. S1 Table. STROBE Statement- checklist of items that should be included in reports of observational studies. S1 Fig. US images Findings- two FAs treated with three relocations. S2 Fig. US images Findings- multiple bilateral FAs.
(PDF)

S2 File Supplementary material Datatable – Datatable used for statistical analysis. (XLSX)

## Abbreviations

AS: Analysis set
BMI: Body mass index
CA: Cryoablation
cm: Centimeter
FA: Fibroadenoma
IQR: Interquartile range
kg: Kilogram
LOESS: Locally estimated scatterplot smoothing
Min: Minimum
Max: Maximum
N: Number of patients
SAS: Safety analysis set
SD: Standard deviation
US FDA: United States Food and Drug Administration
US: Ultrasound
